# Does productivity influence priority setting? A case study from the field of CVD prevention

**DOI:** 10.1186/1478-7547-6-6

**Published:** 2008-03-17

**Authors:** Lars Lindholm, Emil Löfroth, Måns Rosén

**Affiliations:** 1 Umeå International School of Public Health and Centre for Populations Studies: Ageing and Living Conditions Programme, Umeå University, Sweden; 2 Centre for Epidemiology, National Board of Health and Welfare, Stockholm, Sweden; 3 The Swedish Council on Technology Assessment in Health Care, Stockholm, Sweden

## Abstract

In this case study, different measures aimed at preventing cardiovascular diseases (CVD) in different target groups have been ranked based on cost per QALY from a health care sector perspective and from a societal perspective, respectively. The innovation in this study is to introduce a budget constraint and thereby show exactly which groups would be included or excluded in treatment or intervention programs based on the two perspectives. Approximately 90% of the groups are included in both perspectives. Mainly elderly women are excluded when the societal perspective is used and mainly middle-aged men are excluded when the health care sector perspective is used. Elderly women have a higher risk of CVD and generally lower income than middle-aged men. Thus the exclusion of older women in the societal perspective is not a trivial consequence since it is in conflict with the general interpretation of the "treatment according to need" rule, as well as societal goals regarding gender equality and fairness. On the other hand, the exclusion of working individuals in the health care perspective undermines a growth of public resources for future health care for the elderly. The extent and consequences of this conflict are unclear and empirical studies of this problem are rare.

## Introduction

Cost-effectiveness analysis is often considered to be a simple and straightforward tool for resource allocation decisions. However, there are many unsolved methodological controversies debated in the literature, such as the choice of perspective. Many economists recommend a societal perspective based on welfare economics rooted in utilitarian philosophy. The goal of society is commonly assumed to be the maximization of utility, irrespective of distribution. A state with a higher sum of utility is always preferred to a state with a lower sum. However, such a framework raises equity concerns and the use of social welfare functions are a possible solution but are still rare in empirical studies.

Adopting the maximization view in the evaluation of health care programs means that all health effects, costs and savings should be considered independently of the identity of the beneficiary and payer. One important but controversial aspect of this is productivity changes as a consequence of health care interventions [1]. From a societal perspective, these productivity changes should always be included in cost-effectiveness analyses, usually in the numerator. However, there are many opponents to this view and their main argument is fairness – it is not fair to include productivity changes as a savings factor in the calculus because it will discriminate against people with low income (women, pensioners, ethnic minorities, immigrants etc.). A response to this critique has been the use of a "standard" income figure, equal for all individuals in full-time employment [1].

In addition to fairness, some argue that the application of the societal perspective in health implies an imbalance because the effect side is only focused on health and therefore it may be considered inconsistent to include all costs. Brower et al [2] describe a responsibility argument. Decision-makers in health care commonly interpret their mandate as maximizing health gains subject to the resources devoted for this purpose. Thus it is natural for them to only be concerned with the costs and savings that affect the specific budget they are responsible for. This in turn causes the analysts to choose a health care sector perspective. Yet, Brouwer et al [2] suggest the adoption of a two-perspective approach as standard.

In addition to the way in which productivity changes are dealt with, there are several important differences between the societal perspective and the health sector perspective, such as their differing treatment of costs incurred by the patient and/or the family and voluntary caring time. In the current study, however, we focus on the issue of productivity changes.

The precise consequences of including or excluding productivity changes depends of course on the specific context, but in general the consequences are that some patients are treated while others are denied treatment (assuming that decision-makers base their decisions on the findings of cost-effectiveness analyses). It is unlikely that exactly the same groups will be denied treatment based on the two perspectives. Despite general awareness of the consequences of the different perspectives, however, quantifications are rare. We have not found any studies attempting to show who will receive and who will be denied treatment in either a societal or health care sector perspective.

In order to make the consequences of the differing perspectives explicit, the whole process of cost-effectiveness analysis must be outlined. This process has been described as:

"Most often, CEA is applied from a societal viewpoint or from the viewpoint of a national health care system. In this formulation, the implied decision-maker is an agent for society at large, and the objective is to achieve the maximum possible health benefit (e.g. life years, or quality-adjusted life years [QALY's]) subject to overall limits on health-care resources. [3]

However, to our knowledge there have been few attempts in the literature to carry out the whole process of CEA (we are aware of only two: [4,5]).

The aims of this case study are:

A. To show exactly which groups will be excluded from treatment based on a health care sector perspective and a societal perspective respectively;

B. Decompose the ratios and examine the opportunity costs (and QALY's) of a health sector perspective.

## Methods

The case used in this study is the prevention of CVD. An intervention is defined as an effort with the purpose of reducing CVD risk in a defined target group and each intervention thus has the capacity to prevent CVD and produce QALYs. At the same time, interventions also consume resources from the available budget. This amount of resources can be considered as a monetary measure of "need". Culyer and Wagstaff [6] suggest a definition of need relevant to economics: "the minimum amount of resources required to exhaust a person's capacity to benefit i.e. the costs necessary to satisfy a need during a certain time period (e.g. one year)".

The cost of applying an intervention in a target group is equal to the number of people in the target group multiplied by the cost per person.

Savings are based on prevented cases of CVD and they are calculated according to the two perspectives – a societal and a health care sector perspective.

The budget is defined as the actual direct cost of the interventions, i.e. the proportion of the set (the "needs") that are financed today.

We use a deterministic model to compute the effect of an intervention. The starting point is a cohort of CVD-free individuals. Every year the cohort is exposed to the risk of suffering a myocardial infarction (MI), suffering a stroke, or death from other causes.

Separate risk functions were used for MI and stroke. The one-year risk of a MI or a stroke is the annual age- and sex-specific incidence adjusted for the difference between the risk factor level in a studied group and the mean risk factor level in the population [7]. To estimate non-fatal and fatal incidences, the Swedish Hospital Patient Discharge Register and the Cause of Death Register were used respectively [8].

The costs included for the interventions are drugs and smoking cessation (table 1), and hospital treatment and production loss relating to manifest disease (table 2). In the societal perspective (table 1), the patient's travel costs as well as their co-payment for drug costs are added. The value of production was estimated as the difference between the annual gross income for the patients with an MI or stroke and the general population. The estimations were based on all patients between 1995 and 1998 in Sweden using the Swedish Hospital Patient Discharge Register, the Cause of Death Register and data of income registered at Statistics Sweden [8,9]. The hospital treatment costs are a result of MI or stroke and are stratified according to the first year and all subsequent following years. The QALY weights are obtained from the literature [10].

**Table 1:** Costs of different interventions (SEK) in two perspectives.

Intervention	Cost (SEK)	
	Health care sector perspective	Societal perspective

Smoking cessation	150	150
Blood pressure reduction	2284	3122
Cholesterol reduction	3641	4600
Blood pressure reduction and smoking cessation	2434	3272
Cholesterol reduction and smoking cessation	3791	4750
Blood pressure reduction and cholesterol reduction	4725	6182
Blood pressure reduction, cholesterol reduction and smoking cessation	4875	6332

**Table 2:** Assumed costs (tSEK) for production losses and hospital treatment, and QALY-weights.

	1st year								Subsequent years							
	Female				Male				Female				Male			

	40–49		50–64		40–49		50–64		40–49		50–64		40–49		50–64	

	MI	S	MI	S	MI	S	MI	S	MI	S	MI	S	MI	S	MI	S

Production losses	60	55	66	66	67	71	68	83	55	70	83	85	70	94	85	113
Hospital treatment	59	72	59	72	59	72	59	72	8	54	8	54	8	54	8	54
QALY-weight	0.75	0.5	0.75	0.5	0.75	0.5	0.75	0.5	0.95	0.75	0.95	0.75	0.95	0.75	0.95	0.75

Three interventions to prevent CVD are included in this study: smoking cessation advice; hypertension drugs; and cholesterol drugs. A single intervention or a combination of two or three may be taken. Thus there are a total of eight different possible intervention strategies and it is necessary to analyse the incremental effects and costs of each.

The health gains of any particular intervention depend, among other things, on the risk of the target group. Therefore, the population is stratified according to risk into 108 groups (table 3).

**Table 3:** Combination of risk factors used in the stratification of the population.

Age	40 – 49, 50 – 59, 60 – 69
Sex	Female, Male
Smoking	Yes, No
Cholesterol	-5,9 mmol/l, 6,0–7,4 mmol/l, 7,5 mmol/l -
Blood pressure	-139 mmHg, 140–179 mmHg, 180 mmHg -

The effect of cholesterol-lowering drugs is assumed to be a 20% reduction in the yearly risk for both MI and stroke [11,12]. The effect of blood pressure lowering drugs is assumed to be a 16% reduction in risk for MI and 38% risk reduction for stroke [13]. The effect of smoking cessation is a 45% reduction in the yearly risk of MI and a 49% reduction in the yearly risk of stroke [14].

We calculated the cost per QALY in two ways, according to two sets of cost components in table 1 and 2, and productivity gains were only included in calculations based on the societal perspective. Budget claims was set as equal to "local needs", which were calculated as the number of persons belonging to certain risk groups (and thus the population that could expect improved health from the intervention) multiplied by the direct intervention cost per person. The county council budget for a certain purpose is equal to the amount of resources currently used for that specific purpose. In total, the resources spent on primary prevention of CVD with drugs and smoking cessation in Västerbotten county council is 36.5 million SEK and this amount is used as a budget constraint in the calculations. All the interventions were ranked, initially according to the cost-effectiveness ratio based on societal costs, and subsequently on the ratio including health care costs only. Thus two different rankings of each intervention up to the budget limit are presented here.

## Results

Altogether 160 combinations of groups and treatments were not dominated and constitute the complete "league-table" in this study. 94 groups would be given the same treatment in both perspectives even if the ranking order differed. Twelve groups (notations A to L) are included either in the societal or the health care sector perspective (table 4). 57 groups were excluded from treatment using the societal perspective and 63 groups using the health care sector perspective. The group A-I contains 4053 individuals and the group J-L 4039, so the treatment costs are equal (figures not shown). In the societal perspective, A to I have ratios equal to or below 54101 SEK/QALY and are included while J to L have ratios equal to or larger than 54855 SEK/QALY. In the health care sector perspective, J to K have the lowest ratios (59413–77047 SEK/QALY) and are included, while A-I have ratios of 79599 SEK/QUALY or greater, and are thus excluded. The main pattern is that older females (J-L) are included in the health care sector perspective only, while primarily younger males and some females (A-I) are included in the societal perspective only.

**Table 4:** Treatment groups either excluded in the societal or health care sector perspective

Intervention	Sex and age	Risk profile	Gained QALYs	Societal perspective			Health care sector perspective		
				Net costs, SEK	Cost/QALY	Included	Net costs, SEK	Cost/QALY	Included

A	M_40_	5.0, 180	6	1	167	Yes	606	101000	No
B	M_40_	S, 6.0, 150	90	434	4822	Yes	7947	88300	No
C	M_40_	S, 7.5, 139	63	1045	16587	Yes	6451	102397	No
D	M_50_	S, 5.0, 150	147	3036	20653	Yes	12194	82952	No
E	M_40_	7.5, 150	68	1772	26059	Yes	7105	104485	No
F	M_50_	S, 6.0, 139	346	13254	38306	Yes	32258	93231	No
G	M_50_	6.0, 150	624	26209	42002	Yes	58805	94239	No
H	F_50_	S,6.0,150	247	13257	53672	Yes	19661	79599	No
I	M_50_	7.5, 139	227	12281	54101	Yes	23964	105568	No
A-I			1812	71289					
J	F_60_	7.5, 150	765	41964	54855	No	45451	59413	Yes
K	F_60_	6.0,150	1541	105851	68690	No	112247	72841	Yes
L	F_60_	S,6.0,139	235	16945	72106	No	18106	77047	Yes
J-L			2541	164760					

M = male

F = female

SEK = Swedish Crowns

5.0, 6.0, 7.5 = cholesterol levels

139, 150, 180 = blood pressure levels

The two perspectives will cause different consequences on the margin measured as gained QALY's and net costs. Comparing A-I with J-L, the former interventions have a 93 million lower net cost (71289 tSEK versus 164760 tSEK), while the latter (J-L) gains 729 more QALY's. From a health maximization point of view, the health care sector perspective must be preferable. From a welfare maximization point of view, the situation is unclear.

## Discussion

This is a case study bearing the inherent limitations regarding generalization. However, only empirical studies can provide the information necessary for a deeper understanding of the potential conflict between the two CEA perspectives. Not even the most convinced advocates for a certain principle are likely to be completely insensitive to the size of the sacrifices one has to make when principles clash.

Our calculations show that the ranking order is sensitive to the choice of perspective but, in general, when a budget constraint is introduced the same groups will receive treatment. Therefore one can say that the choice of perspective is only important for those groups close to the budget line.

The health care sector perspective is more effective in producing health gains. If the calculations are further decomposed, age is a critical factor in several respects:

1. The risk for disease increases with age.

2. The accumulated gains counted as QALYs are larger the younger the person is at the time of the prevented event (ceteris paribus).

3. The cost for a continuous treatment such as hypertension drugs are larger the younger the person is at the time of the initiation of treatment. "Point interventions" such as smoking advice have the same costs independent of age.

4. The accumulated production losses are larger the younger the person is at the time of the prevented event. The production losses normally approach zero soon after retirement.

Points 1–3 above are common for the two perspectives. In the health sector perspective the higher risk and lower treatment costs for old women outweigh the longer duration of the gaining period for younger males. However, in the societal perspective the latter have a higher lifetime income resulting in larger productivity gains in the case of successful prevention and thus a lower net cost. This pattern would be even more pronounced if individuals aged over 60 were included in the study. Initially, our intention was to include individuals up to 70 years of age since they have almost completely left the labour-market, however this proved to be impossible because epidemiological data for that age-group were not available.

In the example used here (CVD) the risk of disease increases sharply with age thereby compensating for declining income in the ratio calcuation. This example is likely to be representative of many diseases since incidence is often positively correlated with age.

Productivity changes is not the only components in the calculations that are controversial from a normative point of view. It has been argued that QALY's are ageist because younger people typically have a longer life expectancy and treatment of younger individuals thus yields more QALYs than similar treatment of older people [15]. The counter argument is best known as the "fair innings" argument [16], which argues that everyone is entitled to a fair innings of life, and the old have had more of their innings than the young. This position receives some support from several empirical studies indicating that people in general want to give priority to the young over the old [17,18]. However, there exist two qualitatively different reasons for this standpoint. One has its roots in equity considerations – the young have lived less than the old. The second is based on efficiency considerations – the benefit to society is larger if priority is given to young people.

One circumstance making this even more complicated is the dependence between the health of the working population and public resources for health and elderly care. Olsen and Richardson [19] investigate this dilemma and argue that most publicly funded health care is based on the principle of "equal access for equal need", meaning that a health gain has the same social value irrespective of the income level of the beneficiaries. Thus it would be wrong to exclude older women. But a dilemma arises if economic evaluations strive to incorporate this principle. On one hand, the fact that a patient's priority depends on his income is in conflict with "equal access for equal need". On the other hand, to disregard increased productivity gains means ignoring increased societal welfare, which is the fundamental core of welfare economics.

Brouwer et al.[2] discuss the conflict between the broad societal perspective and the more narrow perspective of health care decision-makers. In some European countries, decision-makers have a democracy mandate, they are responsible for a certain budget and equity goals are important. This creates tension between the two perspectives and, assuming that the purpose of health economic analyses is to aid decision-makers, one can question if all dollars have the same value. "We conclude that although all costs are equal, in a health economic evaluation, some may be more equal than others." [2 p 347]

To summarize, allocating health care resources often requires a trade off between conflicting principles. An ambition to establish a general balance seems to imply futile efforts. Rather, the balance has to be set from case to case. Baltuseen and Niessen [20] have proposed a multi-criteria analysis for priority setting in health care, and we believe this would be a step towards more appropriate assistance to the decision-makers. It has been argued that an analysis in two perspectives would be a part of such a development, and we agree. However, we want to add that more studies making the consequences of different perspectives visible would be a further step forward. Who will be treated and who will not? Thus we need to involve the cost-effectiveness studies in a budgetary context more often.

## Conclusion

In this case study, roughly the same groups are prioritised for treatment in the two perspectives. The exclusion of old women in the societal perspective is, however, not a trivial consequence from equity or fairness points of view. On the other hand, the exclusion of young working males in the health care perspective decreases, in principle, societal resources available for future health and elderly care. Whether, this is a typical or an infrequent case is not clear because empirical studies of this problem are lacking. We thus demand more "case studies" in order to increase our understanding of the potential conflict between the two perspectives.

## Competing interests

The author(s) declare that they have no competing interests.

## Authors' contributions

LL, EL and MR designed the study. EL made the calculations. LL, EL and MR interpreted the results. LL drafted the manuscript. EL and MR critically revised the manuscript. LL, EL and MR have approved the final version.
